# Draft Genome Sequence of an Acinetobacter baumannii Isolate Recovered from a Horse with Conjunctivitis in Germany

**DOI:** 10.1128/MRA.01128-19

**Published:** 2019-11-27

**Authors:** Gamal Wareth, Mostafa Y. Abdel-Glil, Gernot Schmoock, Ulrike Steinacker, Heike Kaspar, Heinrich Neubauer, Lisa D. Sprague

**Affiliations:** a Friedrich-Loeffler-Institut, Institute of Bacterial Infections and Zoonoses (IBIZ), Jena, Germany; b Federal Office of Consumer Protection and Food Safety (BVL), Berlin, Germany; University of Maryland School of Medicine

## Abstract

Here, we announce the draft genome sequence of Acinetobacter baumannii strain 161514, which was recovered from a horse with conjunctivitis, and determine the genetic basis of its antimicrobial resistance phenotype. The genome has a size of 3,839,365 bp and a G+C content of 38.93% and is predicted to contain 3,529 coding sequences. The isolate belongs to sequence type 462 (ST462) according to the Oxford scheme (Abaumannii1) and to ST46 according to the Pasteur scheme (Abaumannii2).

## ANNOUNCEMENT

Acinetobacter baumannii is a Gram-negative, opportunistic pathogen from the genus Acinetobacter and a member of the family Moraxellaceae. It is a serious and emerging pathogen that causes nosocomial and community-acquired infections in humans, with a high incidence among immunocompromised patients (1). In animals, it is associated with mastitis, wound infections, septicemia, bronchopneumonia, and eye infections. Horses have been reported to develop wound infections, septicemia, and bronchopneumonia, as well as neonatal encephalopathy and eye infections (2).

A. baumannii strain 161514 was isolated in Germany in 2016 from a horse with conjunctivitis. The sample was a part of a resistance monitoring system aimed at collecting resistance data on animal-pathogenic bacteria (GERM-Vet-2016). The sample was cultured on Columbia blood agar at 37°C, and initial microbial species identification was done using matrix-assisted laser desorption ionization–time of flight mass spectrometry (MALDI-TOF MS; Bruker Daltonik GmbH, Bremen, Germany) and PCR by detection of the *bla*_OXA-51-like_ carbapenemase gene (3).

MICs were determined by the microdilution method using the Micronaut MDR large animal 3, MRGN-screening 3, and β-lactamase plates (Merlin Diagnostika GmbH, Bornheim-Hersel, Germany). The susceptibility to the panel of antimicrobial agents was determined as described in the manufacturer’s instructions and interpreted automatically according to the CLSI guidelines (Table 1).

**TABLE 1:** Genetic features of Acinetobacter baumannii isolate 161514, recovered from a horse

Parameter^*a*^	Value
Total no. of reads	1,399,296
Avg read length (range [bp])	250.2 (35–301)
No. of reads after trimming	1,284,782
No. of contigs	22
*N*_50_ (bp)	474,099
Largest contig size (bp)	1,006,808
Genome coverage (×)	108
Genome	
Size (bp)	3,839,365
GC content (%)	38.93
Genetic elements	
Total no. of genes	3,657
Total no. of CDS	3,587
No. of coding genes	3,529
No. of coding CDS	3,529
No. of RNA genes	70
No. of rRNAs (5S, 16S, 23S)	1, 1, 1 (draft)
No. of tRNAs	63
No. of tmRNAs	1
No. of ncRNAs	4
Total no. of pseudogenes	58
MLST results	
Abaumannii1 (Oxford)	ST462 (Oxf_gdhB_59 A>T)
Abaumannii2 (Pasteur)	ST46
Database accession no.	
GenBank	RPDK00000000
BioProject	PRJNA505595
BioSample	SAMN10426731
Genetic resistance determinants	
β-Lactams (%)	*bla*_OXA-104_ (100), *bla*_OXA-203_ (99.88), *bla*_OXA-ADC25_ (95.94), *bla*_ADC-76-1777_ (96.88), *bla*_A1-1063_ (91.64), *bla*_A2-1542_ (97.1), *bla*_MBL-329_ (96.7), *bla*_OXA-203-719_ (99.88)
Efflux pumps	AdaAB, AdaIJK, and AdaFGH
Phenotypic resistance (MIC [mg/liter])	
Ampicillin	16
Azetronam	>16
Cefoperazone	32
Ceftiofur	>4
Cephalothin	>128
Erythromycin	>4
Florfenicol	>128
Fosfomycin	>64
Penicillin G	>32
Spectinomycin	>64
Tiamulin	>64
Tilmicosin	64
Tulathromycin	64

a CDS, coding sequences; tmRNA, transfer-messenger RNA; ncRNA, noncoding RNA; MLST, multilocus sequence typing.

Genomic DNA was extracted according to the manufacturer’s instructions using the Qiagen Genomic-tip 100/G kit (Qiagen, Hilden, Germany) from a single colony grown in LB medium supplemented with 1% glucose at 37°C. The paired-end sequencing library was prepared with the Nextera XT DNA library prep kit (Illumina, Inc., San Diego, CA, USA). The library was sequenced using a MiSeq sequencer (Illumina, Inc.). Raw reads were processed using the shovill pipeline v1.04 (https://github.com/tseemann/shovill) to produce assembled FASTA files of more than 500 bp in length and with a kmer coverage greater than 3 (enabled options, -trim, -minlen 500, -mincov 3). Quality assessment of the assembled data was conducted with QUAST v4.3 using standard settings (4). Genome annotation was performed using PGAP v4.9 and the NCBI database (5). The distribution of genes (including resistance genes) assigned to the SEED subsystems and the initial metabolic reconstruction were analyzed using RAST (6) (Table 1). Multilocus sequence typing (MLST) of the assembled A. baumannii 161514 sequence was performed using both the Oxford and Pasteur schemes (7, 8) (Table 1). The prevalence of antimicrobial resistance (AMR) genes and variants organized by antibiotic resistance ontology phenotype classification was determined with Resistance Gene Identifier (RGI) v4.2.0 (DIAMOND homolog detection) based on the Comprehensive Antibiotic Resistance Database (CARD) v2.0.3 (9). The AMR genes and/or chromosomal mutations in the isolated strain were determined using the SRST2 tool v0.2.0 (10) and the databases ResFinder v3.1 (11) and ARG-ANNOT (12) (Table 1). Default parameters were used for all software tools unless stated differently.

Acinetobacter baumannii is a serious and emerging pathogen that causes nosocomial and community-acquired infections in humans. The data on the impact of A. baumannii strains from (companion) animal origin on human health are still inconclusive. Further comprehensive studies are needed to assess the possible zoonotic risk of this agent for human and animal health.

### Data availability.

The sequence data of A. baumannii 161514 were deposited in the NCBI archive under GenBank accession number RPDK00000000, SRA accession number SRR8191030, BioProject accession number PRJNA505595, and BioSample accession number SAMN10426731.

## References

[1] Sebeny PJ, Riddle MS, Petersen K 2008 *Acinetobacter baumannii* skin and soft-tissue infection associated with war trauma. Clin Infect Dis 47:444–449. doi:10.1086/590568. 18611157

[2] van der Kolk JH, Endimiani A, Graubner C, Gerber V, Perreten V 2019 *Acinetobacter* in veterinary medicine, with an emphasis on *Acinetobacter baumannii*. J Glob Antimicrob Resist 16:59–71. doi:10.1016/j.jgar.2018.08.011. 30144636

[3] Turton JF, Woodford N, Glover J, Yarde S, Kaufmann ME, Pitt TL 2006 Identification of *Acinetobacter baumannii* by detection of the *bla*_OXA-51_-like carbapenemase gene intrinsic to this species. J Clin Microbiol 27:351–353. doi:10.1128/JCM.01021-06. PMC1594603 16891520

[4] Gurevich A, Saveliev V, Vyahhi N, Tesler G 2013 QUAST: quality assessment tool for genome assemblies. Bioinformatics 29:1072–1075. doi:10.1093/bioinformatics/btt086. 23422339 PMC3624806

[5] Tatusova T, DiCuccio M, Badretdin A, Chetvernin V, Nawrocki EP, Zaslavsky L, Lomsadze A, Pruitt KD, Borodovsky M, Ostell J 2016 NCBI Prokaryotic Genome Annotation Pipeline. Nucleic Acids Res 44:6614–6624. doi:10.1093/nar/gkw569. 27342282 PMC5001611

[6] Aziz RK, Bartels D, Best AA, DeJongh M, Disz T, Edwards RA, Formsma K, Gerdes S, Glass EM, Kubal M, Meyer F, Olsen GJ, Olson R, Osterman AL, Overbeek RA, McNeil LK, Paarmann D, Paczian T, Parrello B, Pusch GD, Reich C, Stevens R, Vassieva O, Vonstein V, Wilke A, Zagnitko O 2008 The RAST server: Rapid Annotations using Subsystems Technology. BMC Genomics 9:75. doi:10.1186/1471-2164-9-75. 18261238 PMC2265698

[7] Bartual SG, Seifert H, Hippler C, Luzon MA, Wisplinghoff H, Rodríguez-Valera F 2005 Development of a multilocus sequence typing scheme for characterization of clinical isolates of *Acinetobacter baumannii*. J Clin Microbiol 43:4382–4390. doi:10.1128/JCM.43.9.4382-4390.2005. 16145081 PMC1234098

[8] Diancourt L, Passet V, Nemec A, Dijkshoorn L, Brisse S 2010 The population structure of *Acinetobacter baumannii*: expanding multiresistant clones from an ancestral susceptible genetic pool. PLoS One 5:e10034. doi:10.1371/journal.pone.0010034. 20383326 PMC2850921

[9] Jia B, Raphenya AR, Alcock B, Waglechner N, Guo P, Tsang KK, Lago BA, Dave BM, Pereira S, Sharma AN, Doshi S, Courtot M, Lo R, Williams LE, Frye JG, Elsayegh T, Sardar D, Westman EL, Pawlowski AC, Johnson TA, Brinkman FS, Wright GD, McArthur AG 2017 CARD 2017: expansion and model-centric curation of the comprehensive antibiotic resistance database. Nucleic Acids Res 45:D566–D573. doi:10.1093/nar/gkw1004. 27789705 PMC5210516

[10] Inouye M, Dashnow H, Raven LA, Schultz MB, Pope BJ, Tomita T, Zobel J, Holt KE 2014 SRST2: rapid genomic surveillance for public health and hospital microbiology labs. Genome Med 6:90. doi:10.1186/s13073-014-0090-6. 25422674 PMC4237778

[11] Zankari E, Hasman H, Cosentino S, Vestergaard M, Rasmussen S, Lund O, Aarestrup FM, Larsen MV 2012 Identification of acquired antimicrobial resistance genes. J Antimicrob Chemother 67:2640–2644. doi:10.1093/jac/dks261. 22782487 PMC3468078

[12] Gupta SK, Padmanabhan BR, Diene SM, Lopez-Rojas R, Kempf M, Landraud L, Rolain JM 2014 ARG-ANNOT, a new bioinformatic tool to discover antibiotic resistance genes in bacterial genomes. Antimicrob Agents Chemother 58:212–220. doi:10.1128/AAC.01310-13. 24145532 PMC3910750

